# 3-O-Galloylated Procyanidins from *Rumex acetosa* L. Inhibit the Attachment of Influenza A Virus

**DOI:** 10.1371/journal.pone.0110089

**Published:** 2014-10-10

**Authors:** Andrea Derksen, Andreas Hensel, Wali Hafezi, Fabian Herrmann, Thomas J. Schmidt, Christina Ehrhardt, Stephan Ludwig, Joachim Kühn

**Affiliations:** 1 Institute of Pharmaceutical Biology and Phytochemistry, University of Münster, Münster, Germany; 2 Institute of Medical Microbiology - Clinical Virology, University Hospital Münster, Münster, Germany; 3 Institute of Molecular Virology, University of Münster, Münster, Germany; University of California, San Francisco, United States of America

## Abstract

Infections by influenza A viruses (IAV) are a major health burden to mankind. The current antiviral arsenal against IAV is limited and novel drugs are urgently required. Medicinal plants are known as an abundant source for bioactive compounds, including antiviral agents. The aim of the present study was to characterize the anti-IAV potential of a proanthocyanidin-enriched extract derived from the aerial parts of *Rumex acetosa* (RA), and to identify active compounds of RA, their mode of action, and structural features conferring anti-IAV activity. In a modified MTT (MTT_IAV_) assay, RA was shown to inhibit growth of the IAV strain PR8 (H1N1) and a clinical isolate of IAV(H1N1)pdm09 with a half-maximal inhibitory concentration (IC_50_) of 2.5 µg/mL and 2.2 µg/mL, and a selectivity index (SI) (half-maximal cytotoxic concentration (CC_50_)/IC_50_)) of 32 and 36, respectively. At RA concentrations>1 µg/mL plaque formation of IAV(H1N1)pdm09 was abrogated. RA was also active against an oseltamivir-resistant isolate of IAV(H1N1)pdm09. TNF-α and EGF-induced signal transduction in A549 cells was not affected by RA. The dimeric proanthocyanidin epicatechin-3-O-gallate-(4β→8)-epicatechin-3′-O-gallate (procyanidin B2-di-gallate) was identified as the main active principle of RA (IC_50_ approx. 15 µM, SI≥13). RA and procyanidin B2-di-gallate blocked attachment of IAV and interfered with viral penetration at higher concentrations. Galloylation of the procyanidin core structure was shown to be a prerequisite for anti-IAV activity; *o*-trihydroxylation in the B-ring increased the anti-IAV activity. *In silico* docking studies indicated that procyanidin B2-di-gallate is able to interact with the receptor binding site of IAV(H1N1)pdm09 hemagglutinin (HA). In conclusion, the proanthocyanidin-enriched extract RA and its main active constituent procyanidin B2-di-gallate protect cells from IAV infection by inhibiting viral entry into the host cell. RA and procyanidin B2-di-gallate appear to be a promising expansion of the currently available anti-influenza agents.

## Introduction

Influenza A and B viruses (IAV, IBV) circulating in the human population are responsible for seasonal epidemics of varying extent. At present, the global annual disease burden of seasonal influenza is estimated to be 1 billion infections, 3 to 5 million of severe infections, and 300 000 to 500 000 fatalities. Without doubt, vaccination remains the most important strategy for prophylaxis and control of seasonal influenza [1]. Although predominantly associated with mild symptoms of upper respiratory tract infection, the first pandemic of the 21st century caused by IAV(H1N1)pdm09 impressively demonstrated the global health risks associated with IAV. Ongoing zoonotic infections with avian IAV(H5N1) and (H7N9) in the human population underscore the permanent threat of pandemic outbreaks, of which the “Spanish flu” pandemic of 1918–19 with an estimated number of 50 million deaths world-wide has been the most devastating [2].

Two classes of antiviral drugs have been licensed for the treatment and prophylaxis of influenza [3]. Matrix protein inhibitors, such as amantadine and rimantadine, inhibit viral uncoating. They are ineffective against IBV and are currently not recommended for the treatment of IAV infections due to high levels of resistance [4]. Neuraminidase inhibitors (NAI), such as oseltamivir and zanamivir, inhibit the release of virus progeny from infected cells and viral spread, are effective against IAV and IBV and have been licensed for first-line therapy of influenza. Although the vast majority of currently circulating IAV(H3N2) and (H1N1)pdm09 is sensitive to oseltamivir, the wide-spread use of oseltamivir has led to a high level of IAV(H1N1) resistance in 2008–9 [3], [5]. In IAV(H1N1)pdm09 resistance against oseltamivir is almost exclusively caused by a single amino acid exchange (H275Y) in the neuraminidase [6]. Recently, two novel NAIs have been approved for the treatment of influenza, peramivir and laninamivir octanoate, the latter being effective also against oseltamivir-resistant influenza virus strains [3], [7]. Since monotherapy with each of the NAIs currently licensed may eventually lead to the selection of resistant virus, drug combinations directed against different molecular targets of influenza virus may be a promising strategy to delay the development of resistance and to achieve synergistic effects. Thus, novel viral targets, antiviral agents and therapeutic strategies such as inhibitors of the viral RNA polymerase complex and broadly neutralizing antibodies should be developed and utilized for the treatment and prophylaxis of influenza [8], [9].

Medicinal plant extracts with anti-IAV activity have been described in many publications [10]–[12]. Although in most plant-derived preparations active compounds and structure-activity relationships remain to be elucidated, polyphenols have been frequently identified to be the antiviral principle in plant extracts [13]. In particular, the broad antiviral and antimicrobial activity of green tea and its components has received much attention [14], [15]. In green tea and a number of other polyphenol-rich plant extracts, catechins and proanthocyanidins, a subgroup of polyphenols derived from oligomerized flavan-3-ols, were found to exert antiviral effects against influenza viruses and other enveloped and non-enveloped viruses [16]–[20]. Recently, we have shown inhibition of viral attachment of herpes simplex virus type-1 by proanthocyanidin-enriched extracts from *Rumex acetosa* L. (Polygonaceae) and *Myrothamnus flabellifolia* Welw. [21], [22]. Extracts from *R. acetosa* are a component of modern phytotherapeutical preparations with nationally registered drug status in Europe, and are used in the treatment of acute and chronic respiratory viral infections [23].

Aim of the present study was to investigate the anti-IAV activity of the *R. acetosa* extract (RA) *in vitro*, to identify relevant compounds and structural requirements for anti-IAV activity and to characterize their mode of action. Our results show that RA strongly inhibits growth of IAV by blocking viral entry. The dimeric, digalloylated procyanidin epicatechin-3-O-gallate-(4β→8)-epicatechin-3′-O-gallate (*syn*. procyanidin B2-di-gallate) was identified as main active principle in RA. Galloylation of the procyanidin backbone was found to be a prerequisite for anti-IAV activity.

## Materials and Methods

### Plant material, extract and isolated compounds of *Rumex acetosa*

**Figure 1:**
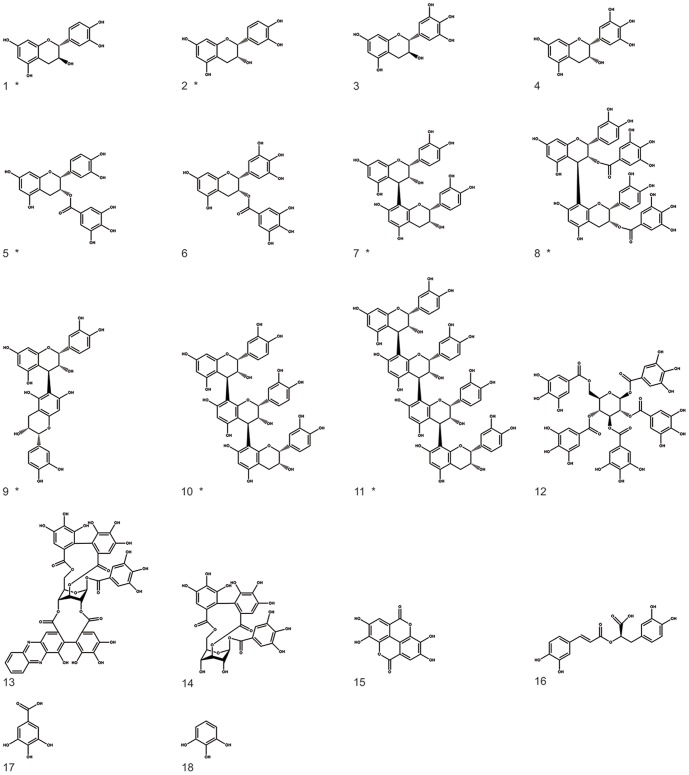
Structural features of flavan-3-ols, oligomeric proanthocyanidins, hydrolyzable tannins, depsides and building blocks of tannins tested for antiviral activity; compounds isolated from *Rumex acetosa* extract RA are marked by asterisk.

Starting materials and preparation of the *Rumex acetosa* L. extract RA have been described recently [21]. Isolation and analytical characterization of proanthocyanidins from RA have been reported by Bicker et al. (2009) [24]. Structural features, sources and purity of flavan-3-ols, oligomeric proanthocyanidins, hydrolyzable tannins, depsides and building blocks of tannins used for antiviral bioassays used in this study are given in Figure 1 and Table 1. Sodium heparin (100,000 IU/g) was purchased from Roth (Karlsruhe, Germany).

**Table 1:** Flavan-3-ols, oligomeric proanthocyanidins, hydrolyzable tannins, depsides and building blocks of tannins tested for antiviral activity.

no.	compound	source	purity^1^	reference
**1**	(+)-catechin monohydrate	Sigma-Aldrich, Steinheim, Germany	≥98%	
**2**	(−)-epicatechin	Sigma-Aldrich, Steinheim, Germany	≥90%	
**3**	gallocatechin	IPBP^2^, Münster, Germany	99%	[51]
**4**	epigallocatechin	IPBP, Münster, Germany	≥80%	[51]
**5**	epicatechin-3-O-gallate	IPBP, Münster, Germany	≥95%	[24]
**6**	epigallocatechin-3-O-gallate	Chengdu Biopurify Phytochemicals Ltd, Chengdu, China	≥95%	
**7**	epicatechin-(4β→8)-epicatechin (*procyanidin B2*)	IPBP, Münster, Germany	97%	[24]
**8**	epicatechin-3-O-gallate-(4β→8)-epicatechin-3′-O-gallate (*procyanidin B2-di-gallate*)	IPBP, Münster, Germany	91%	[24]
**9**	epicatechin-(4β→6)-epicatechin (*procyanidin B5*)	IPBP, Münster, Germany	99%	[24]
**10**	epicatechin-(4β→8)-epicatechin-(4β→8)-epicatechin (*procyanidin C1*)	IPBP, Münster, Germany	97%	[24]
**11**	epicatechin-(4β→8)-epicatechin-(4β→8)-epicatechin-(4β→8)-epicatechin *(procyanidin D1*)	IPBP, Münster, Germany	100%	[24]
**12**	1,2,3,4,6-penta-O-galloyl-β-d-glucose (PGG)	IPBP, Münster, Germany	≥95%	[37]
**13**	geraniin	IPBP, Münster, Germany	≥95%	[38]
**14**	corilagin	IPBP, Münster, Germany	≥95%	[38]
**15**	ellagic acid	Roth, Karlsruhe, Germany	≥95%	
**16**	rosmarinic acid	Sigma-Aldrich, Steinheim, Germany	97%	
**17**	gallic acid monohydrate	Roth, Karlsruhe, Germany	≥97%	
**18**	pyrogallol	Merck, Darmstadt, Germany	≥95%	

1 purity was determined by quantitative HPLC (area %),

2 IPBP: Institute of Pharmaceutical Biology and Phytochemistry.

### Cells and viruses

MDCK II cells (canine kidney cells) were propagated in minimal essential medium (MEM; Biochrom, Berlin, Germany) supplemented with 5% fetal calf serum (FCS; Biochrom), 2 mM l-glutamine (Sigma-Aldrich), non-essential amino acids (1×) and 100 µg/mL penicillin/streptomycin (Biochrom). A549 cells (human lung epithelial cells) were grown in DMEM (PAA Laboratories, Pasching, Austria), supplemented with 10% FCS and 100 µg/mL penicillin/streptomycin. Cytotoxicity, antiviral and penetration assays (see below) were performed using serum-free media.

The influenza A virus laboratory strain A/Puerto Rico/8/34 (PR8), and three clinical isolates of IAV(H1N1)pdm09, i.e., A/Nordrhein-Westfalen/172/09 (NRW172), A/Nordrhein-Westfalen/173/09 (NRW173) [25] and isolate 1/09 (I1) obtained at the Institute of Medical Microbiology – Clinical Virology, University Hospital Münster, were propagated in embryonated chicken eggs. Viral stocks were prepared as described elsewhere [26].

The number of infectious particles in viral stocks was assessed by plaque titration. MDCK II cells were infected with serial dilutions of IAV in PBS containing 0.21% bovine albumin (MP Biochemicals, Eschwege, Germany), 100 U/mL penicillin (Biochrom), 100 µg/mL streptomycin (Biochrom), 230 µmol/L MgCl_2_ (Roth) and 514 µmol/L CaCl_2_ (Roth) for 30 min. at 37°C (500 µL/well). After discarding the inoculum, cells were washed with PBS and covered with 2 mL of overlay medium (MEM [Gibco, Life Technologies, Darmstadt, Germany] containing 100 U/mL penicillin, 100 µg/mL streptomycin, 0.21% NaHCO_3_ [Gibco], 0.01% DEAE-dextran hydrochloride [Sigma-Aldrich], 0.21% bovine albumin, 232 µmol/L MgCl_2_, 518 µmol/L CaCl_2_, 0.00061 ‰ trypsin/829 nmol/L EDTA [Biochrom] and 0.62% Avicel type RC-591 NF [FMC BioPolymer, Philadelphia, PA, USA]). After 48 h of incubation at 37°C, overlay medium was discarded, cells were washed with PBS, fixed with 3.7% formaldehyde for 10 min. and stained with 0.1% crystal violet for 15 min. Subsequently, virus plaques were counted and the infectious titer (pfu/mL) was calculated.

### Cytotoxicity assay, antiviral assays

#### Cytotoxicity assay

The effect of RA and its components on the proliferation of MDCK II cells was determined in 96-well plates (TPP, Trasadingen, Switzerland) using the MTT assay [27] essentially as described by Gescher et al. (2011) [21] with the exception that samples were incubated at 37°C for 1 h prior to addition to cells and remained on the cells for 48 h. The cytotoxic concentration of RA or its components which reduced the cells' viability by 50% (IC_50_) was determined from dose-response curves. The untreated control was arbitrarily set as 100%.

#### MTT_IAV_ assay

The inhibitory effects of RA and other test compounds on the cytopathic effect induced by IAV replication was determined in a MDCK II cell-based assay measuring cell viability by MTT stain (MTT_IAV_ assay) [28]. An inoculum of 1×10^4^ pfu IAV/well (corresponding to a multi plicity of infection of 0.1) was used to infect 96-well plates. All incubation steps were performed with serum-free MEM. In the elementary assay, IAV was pre-incubated with test compounds for 1 h at 37°C and subsequently MDCK II cells were incubated with this RA/IAV mixture for 48 h. In modified assays, either the test compound/IAV mixture was removed from the cells after 60 min., or cells were pre-incubated with test compounds alone for 1 h prior to infection with IAV, or test compounds were added to the cells following a 1 h infection period with IAV.

The antiviral activity was calculated according to the following formula [29]:
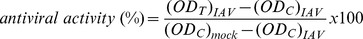



(OD_T_)_IAV_ represents the optical density of cells, which were infected by IAV (index: IAV) and treated with RA. (OD_C_)_IAV_ corresponds to the optical density measured for the untreated IAV-infected cells and (OD_C_)_mock_ is the optical density of untreated, mock-infected cells. The antiviral dose of RA which protected the cells by 50% was defined as the 50% inhibitory concentration (IC_50_).

#### Plaque reduction assay

IAV was incubated with antiviral compounds for 1 h at 37°C, both diluted in PBS containing 100 U/mL penicillin, 100 µg/mL streptomycin, 230 µmol/L MgCl_2_ and 514 µmol/L CaCl_2_. MDCK II cells, cultivated in 12-well culture plates (Greiner Bio-One, Frickenhausen, Germany), were washed with PBS and infected with 300 µL/well IAV/RA-suspension (100 pfu/well). After 30 min. of incubation, the inoculum was removed, 1 mL of overlay-medium without bovine albumin was added and the plates were cultivated for 72 h at 37°C. Subsequently, cells were stained as described above, virus plaques were counted and antiviral activity was calculated by the following formula [21]:
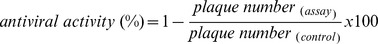



#### Penetration assay

The effect of extract RA and antiviral compounds on viral penetration was determined by a modified plaque reduction assay. In contrast to the basic assay, cells were treated with RA after virus attachment to the cell surface. Penetration of IAV during the attachment and treatment phase was prevented by strictly performing all steps at 4°C.

MDCK II cells, cultivated to 95% confluence in 12-well culture plates, were pre-cooled to 4°C for 15 min. and washed with PBS. 600 pfu IAV, diluted in PBS (400 µL/well) containing 100 U/mL penicillin, 100 µg/mL streptomycin, 230 µmol/L MgCl_2_ and 514 µmol/L CaCl_2_, were allowed to attach to the cells. After 20 min. the inoculum was removed, cells were washed with PBS, PBS containing a 2-fold serial dilution of RA was added and cells were incubated for another 30 min. at 4°C. Before shifting culture plates to 37°C for initiation of viral penetration, cells were washed with PBS and covered with serum-free cultivation medium (see above). Following 30 min. incubation at 37°C, medium was removed and cells were treated with low pH citrate buffer (135 mM NaCl, 10 mM KCl, 40 mM citric acid, pH 3.0) for 15 s to stop penetration and inactivate attached, non-penetrated virions. Low pH buffer was removed by washing twice with PBS, and overlay medium was added. Further cultivation and quantitation of plaques was performed as described above. Mock-treatment of attached virus and inactivation of attached mock-treated virus by low pH citrate buffer immediately prior to the 37°C shift served as controls.

### Hemagglutination inhibition test (HIT)

Twofold serial dilutions (25 µL) of test compounds in PBS and 4 hemagglutinating units (HU) of IAV (25 µl) were mixed carefully in 96-well plates with U-shaped bottom (Thermo Fisher Scientific Nunc, Schwerte, Germany). Plates were shaken for 5 min. and incubated for 25 min. at room temperature (RT). 50 µL of a 1.5% suspension of newborn chicken erythrocytes (RBC) in PBS (Labor Dr. Merk & Kollegen, Ochsenhausen, Germany) were added, and plates shaken again. Assays were read following a 2 h incubation period at RT, and the minimum inhibitory concentration (MIC), defined as the highest test compound dilution showing complete inhibition of the agglutination of erythrocytes, was determined. In every assay, a test compound control (compound plus RBC without addition of IAV), and erythrocyte controls (A: IAV plus RBC, without addition of test compound; B: RBC, without addition of test compound or IAV) were included. Test results were accepted if the back titration of IAV revealed 4 HU and the controls yielded correct results.

### Immunoblotting

The effect of RA or test compounds on IAV envelope proteins was analyzed using recombinant purified HA (20 or 50 µg/mL) of influenza virus A/California/07/2009 (H1N1) (Sino Biological, Beijing, China). SDS-PAGE and blotting was performed essentially as described earlier [21]. To detect IAV HA, membranes were incubated with Anti-IAV H1N1 (Swine Flu 2009) HA antibody (dilution 1: 1000; Sino Biological) or QIAexpress Penta-His Antibody (dilution 1: 500; Qiagen, Hilden, Germany) overnight.

### Signal transduction assay

90–100% confluent A549 cells in 6-well culture plates were washed with PBS and pretreated with 100 µg/mL RA for 1 h at 37°C, or left untreated. Subsequently, cells were stimulated with EGF (30 ng/mL, 10 min.; R&D Systems, Minneapolis, MN, USA) or TNF-α (20 ng/mL, 30 min.; Sigma-Aldrich) in the presence of RA, or left untreated. Cells were washed with PBS twice and lysed with radioimmunoprecipitation assay buffer (25 mM Tris-HCl [pH 8; Roth], 137 mM NaCl [Merck], 10% glycerol [MP Biomedicals, Illkirch, France], 0.1% SDS [Roth], 0.5% DOC [Roth], 1% octylphenoxypolyethoxyethanol [IGEPAL; Sigma-Aldrich], 2 mM EDTA [pH 8; Roth], 50 mM sodium glycerophosphate [Merck Millipore, Billerica, MA, USA], 20 mM TSPP [Roth], plus 1 tablet cOmplete mini [Roche Diagnostics, Mannheim, Germany] per 10 mL buffer) for 45–60 min. at 4°C. Lysates were cleared by centrifugation, and protein content was quantified by the Bradford method. Briefly, 1 mL of 1: 5 diluted protein assay dye reagent concentrate (Bio-Rad Laboratories, Hercules, CA, USA) was added to 5 µL supernatant, absorption at 600 nm was determined and protein contents were adjusted to identical levels. Protein expression was analyzed by SDS-PAGE and immunoblot as described above. For protein detection Anti-ERK1/2 (pT202/pY204) antibody (dilution 1: 1000; BD, Franklin Lakes, NJ, USA) or Phospho-NF-κB p65 (Ser536)(93H1) antibody (dilution 1: 1000; Cell Signaling Technology, Danvers, MA, USA) was employed. Loading controls were performed with Anti-α-Tubulin (Clone DM 1A, dilution 1: 500; Sigma-Aldrich) or Anti-β-Actin (Clone AC-15, dilution 1: 1000; Sigma-Aldrich).

### Statistical analysis

Data represent the means ±SD of at least three independent experiments. Statistical significance was evaluated by a two-tailed one sample *t*-test. A P value of <0.05 indicated a statistically significant difference.

### *In silico* protein-ligand docking

For *in silico* analyses the HA of influenza virus A/California/04/2009 (H1N1) [30] (protein data base ID 3LZG) was used. HA of A/California/04/2009 (H1N1) is closely related to HA of the vaccine strain A/California/07/2009 (H1N1) and HAs of IAV(H1N1)pdm09 strains circulating in the post-pandemic era in Europe and Asia [31], [32]. Epicatechin (**2**), epigallocatechin-3-O-gallate (EGCG) (**6**), procyanidin B2 (**7**) and procyanidin B2-di-gallate (**8**) were docked to the HA of influenza virus *in silico* by the software Molecular Operating Environment (MOE) version 2011.10 (Chemical Computing Group, Montreal, Canada). After identifying potential binding sites at HA with the MOE module “Site Finder”, the test compounds were docked into the 30 cavities with the best PLB (propensity for ligand binding) score using the MMFF94× force field as implemented in MOE. The flexible docking method (induced fit, i.e. both the ligand and the protein binding site were treated as flexible) was applied. The best score of each cavity-compound pair was compared to the best score of the remaining 29 cavities for each of the four compounds. The docking pose represents the best geometry (lowest score) of all investigated orientations of all compounds with respect to all cavities taken into account.

## Results and Discussion

### *Rumex acetosa* extract RA specifically inhibits IAV-infection in cell culture

**Figure 2:**
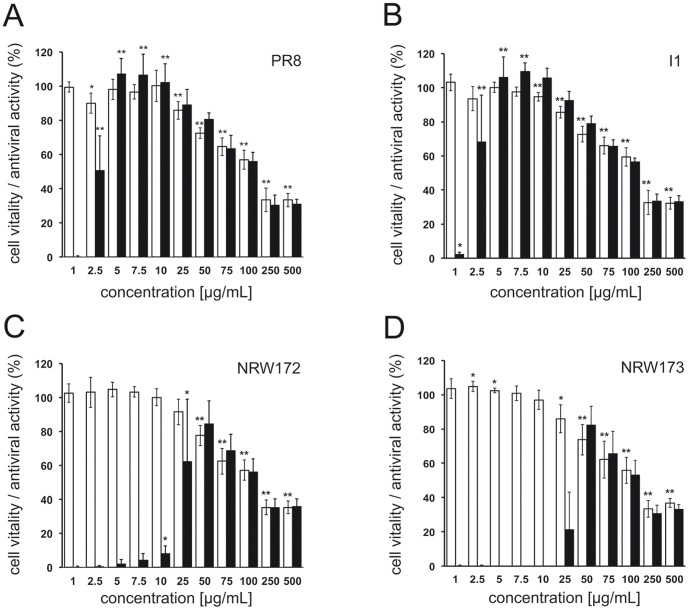
Antiviral and cytotoxic activity of RA on MDCK II cells. 1×10^4^ pfu IAV/well in serum-free medium (antiviral activity, black bars) or serum-free medium (cytotoxic activity, white bars) were incubated with RA at different concentrations indicated for 1 h at 37°C. 48 h after adding the reaction mixtures to 96-well plates, the antiviral activity and cell vitality were determined by MTT_IAV_ assay and cytotoxicity assay, respectively. The following IAV laboratory strains and isolates were used: (A) laboratory strain PR8 [A/Puerto Rico/8/34], (B) clinical isolate I1 [A(H1N1)pdm09], (C) clinical isolate NRW172 [A(H1N1)pdm09], (D) clinical isolate NRW173 [A(H1N1)pdm09]. Values represent mean ±SD of ≥3 independent experiments. * p<0.05, ** p<0.01 (two-tailed, unpaired Student's t-test). Statistical significance of antiviral activity was calculated for nontoxic concentrations only (A: 1 to 10 µg/mL, B: 1 to 7.5 µg/mL, C: 1 to 25 µg/mL, D: 1 to 10 µg/mL).

**Figure 3:**
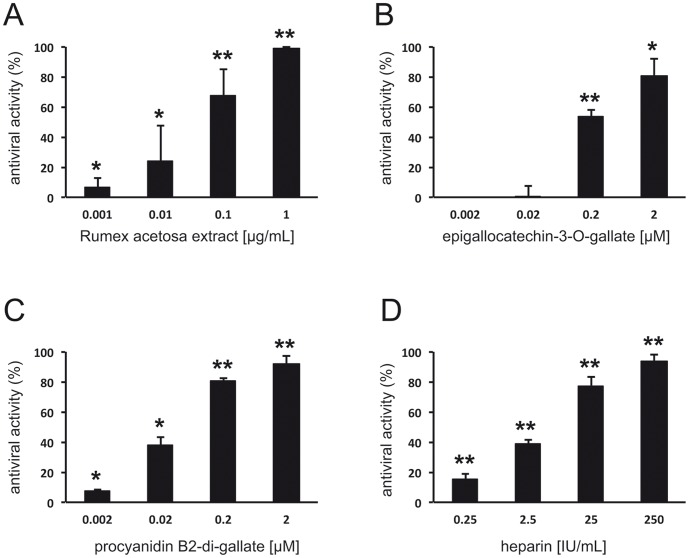
Reduction of IAV plaque formation by the *Rumex acetosa* extract RA (A), epigallocatechin-3-O-gallate (6) (B) and procyanidin B2-digallate (8) (C). IAV and test compounds were co-incubated for 1 h at 37°C prior to the addition to MDCK II cells. Heparin served as positive control (D). Values (% of plaque reduction) ±SD relate to the respective mock-treated controls ( = 100%). * p<0.05, ** p<0.01 (two-tailed, unpaired Student's t-test).

Extract RA and its constituents were screened for anti-IAV-activity by single cycle, MDCK II cell-based MTT_IAV_ assay. Depending on the IAV isolate, the screening window coefficient Z′ of the MTT_IAV_ assay ranged from approx. 0.6 to 0.63, indicating that this assay is well suited to detect inhibitors of IAV entry and replication [28], [33]. Extract RA exhibited 100% antiviral activity against IAV PR8 at concentrations>5 µg/mL with an IC_50_ of 2.5 µg/mL. At extract concentrations≥25 µg/mL a dose-dependent, increasing reduction of cell vitality was observed. The CC_50_ of extract RA was determined to be approximately 80 µg/mL which corresponds to a selectivity index (SI = CC_50_/IC_50_) of 32 (Figure 2A). Almost identical data were found for the clinical isolate I1 of IAV(H1N1)pdm09 with an IC_50_ of 2.2 µg/mL, and a SI of 36 (Figure 2B). The results obtained by MTT_IAV_ assay were corroborated by plaque reduction assay. At a concentration of 100 ng/mL extract RA reduced plaque formation of IAV(H1N1)pdm09 I1 in a highly significant manner by 67%, at 1 µg/mL by 100% (Figure 3).

The antiviral effect of extract RA was tested in two additional clinical isolates of IAV(H1N1)pdm09 obtained in consecutive samples of a patient with acute respiratory distress syndrome. The oseltamivir-sensitive isolate NRW172 was obtained early after hospitalization, the oseltamivir-resistant isolate NRW173 was isolated after completion of oseltamivir therapy. Extract RA inhibited growth of NRW172 and NRW173 with similar efficiency. The IC_50_ values determined for NRW172 (19 µg/mL) and NRW173 (37 µg/mL) in MTT_IAV_ assay were approximately 10-fold higher as observed in IAV PR8 and IAV I1 (Figure 2C, D). Previous work indicated that a high protein load of samples may reduce the antiviral activity of extract RA [22]. Since stocks of IAV NRW172 (6.6×10^6^ pfu/mL) and NRW173 (8.3×10^6^ pfu/mL) contained significantly lower virus titers than stocks of IAV PR8 (3.2×10^8^ pfu/mL) and I1 (3.4×10^7^ pfu/mL), inhibitory effects of residual allantoic fluid on the anti-IAV activity of extract RA were studied. Retesting IAV I1 diluted to 6.6×10^6^ pfu/mL in allantoic fluid of a noninfected egg led to an approx. four-fold increase in the IC_50_ of extract RA (8.2 µg/mL) (Figure S1). Thus, inhibitory effects of residual allantoic fluid on the anti-IAV activity of extract RA appear to account for the differences in IC_50_ values observed in MTT_IAV_ assay. Accordingly, the consistently lower IC_50_ values observed in plaque reduction assay are most likely due to higher dilution of virus stocks during incubation with extract RA. Whether strain specific factors also determine the susceptibility of IAV to extract RA as observed for a polyphenolic extract of *Pelargonium sidoides* DC [34] remains to be clarified.

### Structure-activity relationship: epicatechin-3-O-gallate-(4β→8)-epicatechin-3′-O-gallate (procyanidin B2-di-gallate) (8) is responsible for the antiviral activity of RA

The lead compounds in extract RA have been recently described to be flavan-3-ols and oligomeric proanthocyanidins [24]. To pinpoint the plant secondary products responsible for the antiviral effect of the extract, the dominant proanthocyanidins isolated from extract RA were tested for antiviral effects against IAV I1 and cytotoxicity (Table 2) (for numbering of compounds compare Table 1) at concentrations of 2, 20 and 200 µM, respectively, by MTT_IAV_ and cytotoxicity assay. Additionally EGCG (**6**), a known inhibitor of IAV replication from extracts of green tea which is not present in extract RA [17], [24] was included (Table 2).

**Table 2:** Anti-IAV activity and effect on cell vitality of flavan-3-ols and oligomeric proanthocyanidins from *Rumex acetosa* extract RA and structurally related polyphenolic compounds.

no.	compound	cell vitality^1^				anti-IAV activity^2^			
		2 µM	20 µM	200 µM	CC_50_ (µM)	2 µM	20 µM	200 µM	IC_50_ (µM)
**1**	catechin monohydrate	105±3	102±5	119±9	>200	0±0	0±0	0±0	>200
**2**	epicatechin	101±2	104±4	124±20	>200	0±0	0±0	1±1	>200
**3**	gallocatechin	106±5	108±1	75±17	>200	0±0	0±0	66±5	≤156
**4**	epigallocatechin	101±4	106±1	86±17	>200	0±0	0±0	26±22	>200
**5**	epicatechin-3-O-gallate	107±3	106±4	41±2	175	0±0	1±0	38±2	≤258
**6**	epigallocatechin-3-O-gallate	103±4	92±7	62±9	>200	1±1	87±25	60±7	≤12
**7**	procyanidin B2	107±6	108±9	117±8	>200	0±0	0±0	1±1	>200
**8**	procyanidin B2-di-gallate	106±5	90±3	48±4	191	1±1	71±35	46±4	≤15
**9**	procyanidin B5	100±3	84±4	57±4	>200	0±0	0±0	63±4	≤163
**10**	procyanidin C1	104±2	99±4	122±6	>200	0±0	0±0	87±5	123
**11**	procyanidin D1	101±6	86±8	54±9	>200	0±0	1±0	44±7	≤225
**12**	PGG	91±7	57±14	37±3	83	3±3	46±9	35±1	≤22
**13**	geraniin	99±1	107±2	35±3	162	0±0	1±1	25±0	≤388
**14**	corilagin	101±1	103±1	8±1	120	0±-1	1±0	3±2	uncalc.^3^
**15**	ellagic acid	106±1	110±3	108±2	>200	0±1	1±1	4±1	>200
**16**	rosmarinic acic	102±1	103±1	102±1	>200	0±0	0±0	1±1	>200
**17**	gallic acid monohydrate	106±5	107±8	49±18	197	0±1	-1±0	14±11	>200
**18**	pyrogallol	98±4	96±1	43±1	176	0±0	7±3	42±1	≤241

1 cytotoxicity was determined by cytotoxicity assay,

2 antiviral effects against the IAV(H1N1)pdm09 isolate I1 were determined by MTT_IAV_ assay.

3 uncalculable due to strong cytotoxicity.

The monomeric flavan-3-ols catechin (**1**) and epicatechin (**2**) did not show antiviral activity. Trihydroxylation of the B-ring in gallocatechin (**3**) and epigallocatechin (**4**) led to a slightly increased cytotoxicity. Esterification with gallic acid also increased cytotoxicity. Epicatechin-3-O-gallate (**5**) did not show antiviral activity, while EGCG (**6**) exhibited strong activity at concentrations of about 20 µM (estimated SI≥17). These results indicated that an *o*-trihydroxylation in the B-ring and galloylation at position O-3 is responsible for the antiviral effects of flavan-3-ols detected by MTT_IAV_ assay.

Strong antiviral activity was determined for the oligomeric proanthocyanidins in the cases where the epicatechin building blocks are galloylated. While the dimeric epicatechin-(4β→8)-epicatechin (procyanidin B2) (**7**) was inactive, the corresponding di-galloylated procyanidin epicatechin-3-O-gallate-(4β→8)-epicatechin-3′-O-gallate (procyanidin B2-di-gallate) (**8**) exhibited a prominent antiviral activity (IC_50_ of approx. 15 µM) with an SI of about ≥13. It should be noted that the increasing cytotoxicity of active compounds such as procyanidin B2-digallate (**8**) and EGCG (**6**) at high concentrations reduces the extent of cytoprotection against influenza virus detectable by MTT_IAV_ assay. Using the formula given in Materials and Methods to calculate the results of MTT_IAV_ assay, this seemingly reduces the antiviral activity of active compounds at cytotoxic concentrations (200 µM) (Table 2).

Other non-galloylated di- and oligomeric procyanidins from RA with different structural features were inactive. Compared to the epicatechin-(4β→8)-epicatechin (procyanidin B2) (**7**), dimeric epicatechin procyanidins with 4β→6-interflavan linkage such as epicatechin-(4β→6)-epicatechin (procyanidin B5) (**9**) did not show an altered antiviral profile. However, the 4β→6-linked compound (**9**) exerted higher cytotoxicity compared to (**7**) indicating that changes in the planarity of the molecules may significantly influence the effects on cell physiology. The trimeric and tetrameric procyanidins epicatechin-(4β→8)-epicatechin-(4β→8)-epicatechin (procyanidin C1) (**10**) and epicatechin-(4β→8)-epicatechin-(4β→8)-epicatechin-(4β→8)-epicatechin (procyanidin D1) (**11**), respectively, offered no relevant antiviral activity but showed weak cytotoxic effects.

Thus, within the complex mixture of extract RA dominated by flavan-3-ols and proanthocyanidins with different degrees of polymerization and galloylation, the antiviral activity is mostly mediated by galloylated oligomers. The dimeric compound procyanidin B2-di-gallate (**8**) was assessed as the main principle of antiviral activity in extract RA. The content of procyanidin B2-di-gallate (**8**) in extract RA was determined by UHPLC to be 0.96%. The strong antiviral effect of procyanidin B2-di-gallate (**8**) was confirmed by plaque reduction assay (Figure 3). Purified galloylated higher oligomers present in extract RA were not available for antiviral testing, however, most likely are also active against influenza virus. Generally, a higher number of pyrogalloyl moieties, an increased degree of polymerization and a 4β→8 interflavan linkage amplify the anti-IAV activity of polyphenols from extract RA. These findings are in accordance with the results published by De Bruyne et al. (1999) [35] describing similar structural requirements of polyphenols active against HSV and HIV. In addition, trihydroxylation of the B-ring of non-galloylated oligomeric proanthocyanidins has been reported to mediate anti-influenza virus activity [34].

An insignificant anti-influenza activity of the monomeric flavan-3-ols catechin (**1**) and epicatechin (**2**) has been reported earlier [16], [19]. Interestingly, Song et al. (2005) [19] showed that ECG (**5**) a main constituent from green tea strongly inhibited anti-IAV and IBV in cell culture whereas EGC (**4**) exhibited little antiviral activity. Yang et al. (2014) [16] found that procyanidin B2 (**7**) significantly inhibited growth of IAV. This is in contrast to our findings where ECG (**5**) and procyanidin B2 (**7**) were screened negative for anti-IAV activity at noncytotoxic concentrations. Most likely, this reflects differences in the test format used, e.g. MTT_IAV_ assay *vs*. plaque reduction assay and cytopathic effect inhibition assay, respectively. In particular, the assays used by Song et al. (2005) [19] and Yang et al. (2014) [16] imply multi-cycle replication of IAV and thus should also detect inhibitory effects of compounds on late steps of the viral replication cycle, such as assembly, maturation and release as reviewed by Beyleveld et al. (2013) [28]. Accordingly, Song et al. (2005) [19] detected a direct inhibition of the viral neuraminidase activity by ECG (**5**), however, not by EGC (**4**).

A prominent virucidal activity of EGCG (**6**) from green tea has been first reported by Nakayama et al. (1993) [17]. As reported for ECG (**5**), EGCG (**6**) also directly inhibits the viral neuraminidase. In addition to anti-influenza activity, EGCG offers broad anti-infective properties against various viral, bacterial and fungal pathogens as reviewed by Steinmann et al. (2012) [15].

After oral application, proanthocyanidins exhibit a very limited bioavailability as reviewed by Zumdick et al. (2012) [36]. Thus, the oral application of active compounds such as procyanidin B2-di-gallate (**8**) for the systemic treatment of influenza virus infection appears to be inappropriate. As an alternative, the local application of procyanidins in the upper respiratory tract, either by lozenges, chewing gums etc. or by inhaling devices allows the active compounds to directly contact the virus and should be preferred.

Because proanthocyanidins are known to have tannin-like effects it might be assumed that these polyphenols from extract RA nonspecifically inactivate essential viral structural proteins. Therefore we included other polyphenols not being part of extracts from *R. acetosa*, but with known strong astringent activity (Table 1). Pentagalloyl-glucose (PGG) (**12**), a well characterized hydrolyzable tannin [37], showed moderate antiviral activity, however, significant cell toxicity in the MTT_IAV_ assay (Table 2). Also the ellagitannins geraniin (**13**), corilagin (**14**) [38] and ellagic acid (**15**) were inactive at the highest concentration tested (200 µM) (Table 2). When added at concentrations in the millimolar range, ellagic acid (**15**) has been reported to exhibit broad anti-influenza activity *in vitro* and *in vivo* [39]. The depside rosmarinic acid (**16**), known as tannin-like compound, was also inactive. Keeping in mind that also oligomeric procyanidins such as procyanidin B2, C1 or D1 (**7, 10, 11**) are known to interact strongly with proteins in a tannin-like manner, nonspecific denaturing effects do not appear to account for most of the antiviral activity observed for procyanidin B2-di-gallate (**8**). Otherwise, a more potent activity of the hydrolyzable tannins geraniin (**13**) and corilagin (**14**) should have been observed. An exception appears to be PGG (**12**), which exhibited moderate anti-IAV activity in MTT_IAV_ assay with an IC_50_ of 22 µM. This might be due to its flexible structure. In contrast to geraniin (**13**), PGG (**12**) owns the capacity to rotate its galloyl moieties relatively to the glucose. As a result PGG (**12**) may be able to bind more strongly to proteins. In accordance with our results, PGG (**12**) has been recently reported to possess anti-IAV activity at micromolar concentrations and to inhibit viral entry, budding and release [40].

Since only the galloylated compounds (**6**) and (**8**) exhibited prominent antiviral activity, we tested the effect of free gallic acid (**17**) and pyrogallol (**18**), mimicking a trihydroxylated phenyl system. Both compounds, however, showed only moderate antiviral activity yet relevant cytotoxicity at a concentration of 200 µM. Theissen et al. (2014) [41] recently reported that gallic acid (**17**) inhibits reporter gene expression of the recombinant IAV laboratory strain A/Puerto Rico/8/34-NS116-GFP in a multi-cycle assay with an EC_50_ of approx. 50 µM and a SI of approx. 15. Similar to our findings, however, preincubation of IAV(H1N1)pdm09 particles for 2 h with 50 µg/mL (corresponding to 265 µM) gallic acid (**17**) had only little effect on virus replication in A549 cells. Furthermore, gallic acid (**17**) poorly inhibited IAV neuraminidase with an IC_50_ of>500 µM. Thus, the inhibitory mechanism of gallic acid (**17**) on IAV replication remains to be clarified.

### Extract RA affects viral attachment

To identify steps in the viral life cycle that were affected by extract RA, virus and cells were treated with extract RA at different times pre and post infection. If pre-treated IAV was added to cells for 1 h, viral replication was inhibited completely at concentrations of extract RA>10 µg/mL. In contrast, if cells were infected with IAV and extract RA was added after 1 h, no antiviral effect was observed at ≤10 µg/mL, indicating that extract RA does not operate in the post-entry phase (data not shown).

To determine whether extract RA interacts with target molecules of the host cells or of the virus, MDCK II cells were incubated with extract RA for 1 h and subsequently infected with IAV. At concentrations of ≤10 µg/mL this preincubation of the host cells did not result in any antiviral effects (data not shown). This suggests that the anti-IAV activity of extract RA is caused by direct interaction with IAV particles and inhibition of viral entry as shown for a number of polyphenol and tannin-rich plant extracts in earlier reports [17]–[19], [39], [41]–[44].

**Figure 4:**
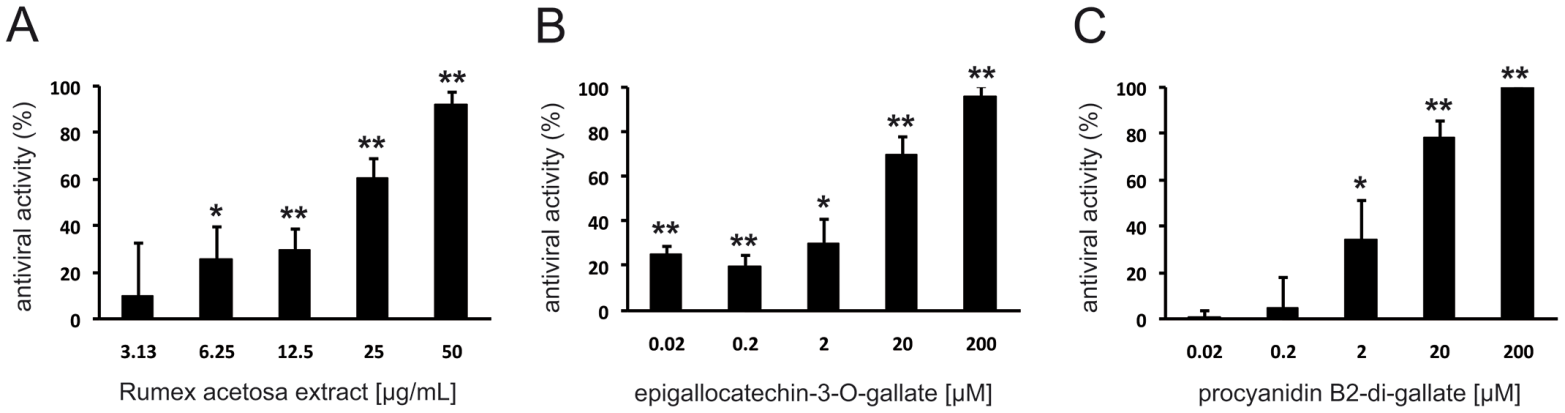
Effect of *Rumex acetosa* extract RA (A), epigallocatechin-3-O-gallate (6) (B) and procyanidin B2-digallate (8) (C) on the penetration of IAV. Effects on the penetration of IAV were determined by a modified plaque reduction assay. Test compounds were added for 30 min. after attachment of IAV to MDCK II cells at 4°C. Values (% of plaque reduction) ±SD relate to the respective mock-treated controls ( = 100%) and represent ≥3 independent experiments. * p<0.05, ** p<0.01 (two-tailed, unpaired Student's t-test).

To reconnoiter the effect of extract RA to inhibit penetration of IAV particles already attached to the cell surface we used a penetration assay. Cells were infected at 4°C, unbound viral particles were removed by washing, extract RA was added at 4°C for 30 min., and penetration was allowed to occur by a temperature shift to 37°C (30 min.) followed by washing with pH 3.0 citrate buffer to inactivate non-penetrated virus. As shown in Figure 4, extract RA also blocks viral penetration. However, in comparison to incubation of IAV with extract RA prior to entry, significantly higher concentrations of extract RA were needed to achieve comparable antiviral effects. Washing of cells with pH 3.0 citrate buffer at 4°C immediately after the adsorption period and prior to shifting the temperature to 37°C completely abrogated plaque formation. These observations suggested that RA affects virus entry primarily by inhibiting viral attachment. Similar results were also obtained with EGCG (**6**) and procyanidin B2-di-gallate (**8**) (Figure 4). As discussed above, the relatively high protein load due to the presence of cells and culture media components may increase the concentration of RA and its active constituents needed to inhibit penetration of IAV already attached to the cell surface. When added after the infection of MDCK cells, high concentrations of green tea extract and EGC (**4**) have been reported to affect the early phase of influenza virus infection, possibly by interference of the polyphenolic compounds with the acidification of endosomes [18].

### RA and galloylated oligomeric procyanidins interact with IAV hemagglutinin

Data presented above suggested that extract RA, EGCG (**6**) and procyanidin B2-di gallate (**8**) may interfere with the sialic acid receptor binding function of the viral HA. Therefore, effects on HA-mediated attachment of IAV to the cell surface were further investigated in a hemagglutination inhibition assay. Using four hemagglutinating units of IAV(H1N1)pdm09 I1 in allantoic fluid (5.5×10^7^ pfu/mL) to agglutinate chicken erythrocytes, pretreatment of the IAV suspension with extract RA inhibited erythrocyte agglutination at a minimum inhibitory concentration of 156 µg/mL (Table 3). At higher concentrations, hemagglutination reappeared due to direct agglutination of erythrocytes by extract RA. By serial dilution of extract RA in PBS the minimal concentration needed to agglutinate erythrocytes in the absence of IAV was determined to be 156 µg/mL. Thus, treatment of IAV with extract RA appears to directly interfere with the cell surface receptor-binding function of IAV HA. Procyanidin B2-di-gallate (**8**) did not inhibit IAV-mediated hemagglutination, however, was able to directly agglutinate erythrocytes at a concentration≥39 µM. In accordance to Theissen et al. (2014) [41] EGCG (**6**) showed no inhibitory effect on IAV-mediated hemagglutination, however, directly agglutinated erythrocytes (Table 3). None of the test compounds induced hemolysis (data not shown). Strong, IAV-strain specific differences in the concentrations of EGCG (**6**) needed to inhibit hemagglutination have been reported earlier [19], and may account for the failure to detect inhibitory effects of procyanidin B2-di-gallate (**8**) and EGCG (**6**) on IAV(H1N1)pdm09 induced hemagglutination.

**Table 3:** Effect of *Rumex acetosa* extract RA and single compounds on IAV-mediated hemagglutination.

compound^1^	MIC	direct agglutination	highest concentration tested
*Rumex acetosa* extract	156 µg/mL	156 µg/mL	10 mg/mL
epigallocatechin-3-O-gallate (**6**)	n.d.^2^	156 µM	5 mM
procyanidin B2-di-gallate (**8**)	n.d.	39 µM	5 mM

1 compounds are numbered as given in Table 1,

2 n.d.: not detectable.

**Figure 5:**
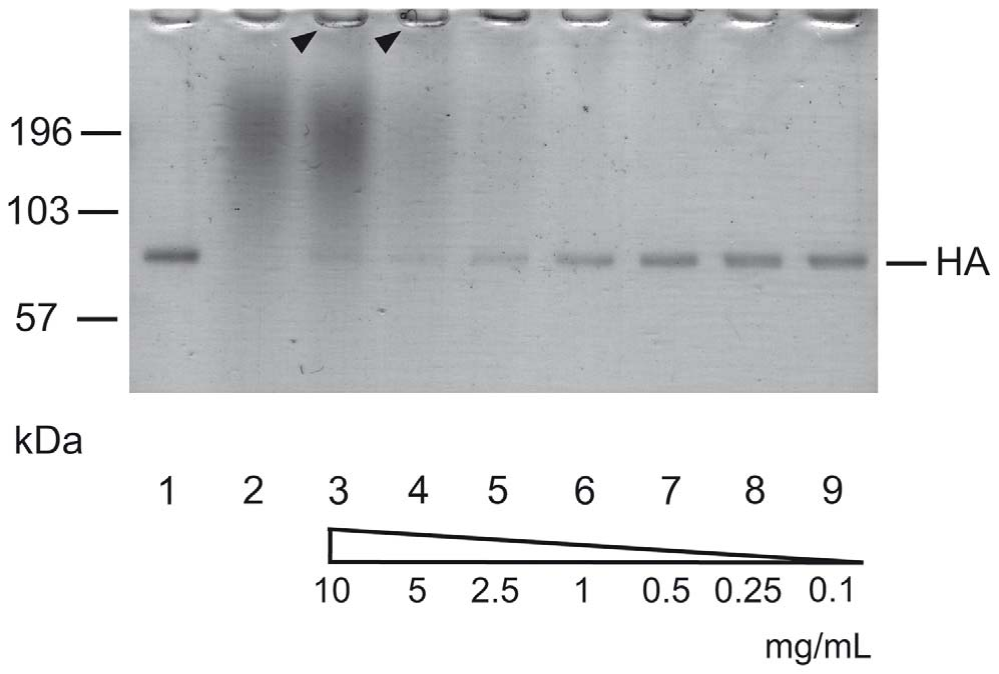
Effect of RA on the electrophoretic mobility of recombinant soluble HA. Mock-treated HA (lane 1), RA (10 mg/mL) (lane 2), and HA treated with RA (0.1 to 10 mg/mL) as indicated for 1 h (lanes 3 to 9) were loaded onto 10% bis-tris SDS-PAGE gels and analyzed by Coomassie-staining. The positions of molecular weight marker (mwm) and HA are indicated. HA conglomerates in the gel pockets are marked by arrowhead.

In addition, the physical interaction of extract RA and its active compounds with recombinant, soluble HA was studied by SDS-PAGE and immunoblotting. Incubation of HA with high concentrations of extract RA, i.e. 2.5 to 10 mg/mL, for 1 h led to the almost complete disappearance of the 75 – 85 kDa HA-specific band in SDS-PAGE (Figure 5) and abrogated reactivity of HA with an HA-specific monoclonal antibody in immunoblotting (data not shown). Extract RA-treated HA appeared to be retained in the gel pockets, most likely due to the formation of large, electrophoretically immobile complexes. At lower concentrations, i.e. 1 to 0.1 mg/mL, extract RA had no effect on the electrophoretic mobility and immunoreactivity of HA, respectively. Taking into consideration that the IAV-specific IC_50_ value of extract RA in MTT and plaque reduction assay is approximately 100 to 1,000-fold lower, this finding supports the conclusion that most of the anti-IAV activity of extract RA is not due to non-specific tannin-like effects on viral proteins.

**Figure 6:**
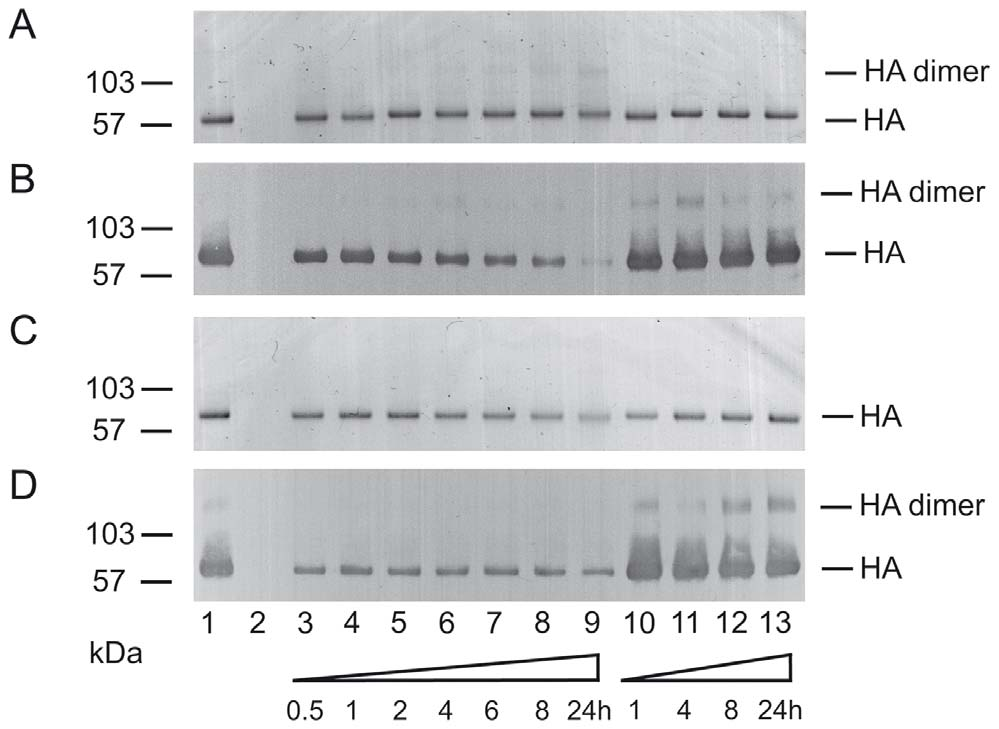
Effect of EGCG (6) (A, B) and procyanidin B2-di-gallate (8) (C, D) on electrophoretic mobility and detection of HA by immunoblotting. Recombinant soluble HA was either mock-treated (lanes 1), incubated with EGCG (6) (2.18 mM) or procyanidin B2-di-gallate (8) (1.13 mM) dissolved in PBS for the times indicated (lanes 3 to 9) or incubated with PBS only (lanes 10 to 13); EGCG (6) (2.18 mM) and procyanidin B2-di-gallate (8) (1.13 mM) incubated in the absence of HA served as control (lanes 2). Figure 6A, C: Coomassie-stained SDS-PAGE. Figure 6B, D: Detection of HA by immunoblot using a penta-His-specific monoclonal antibody. The expected position of monomeric (approx. 75 kDa) and dimeric HA (approx. 150 kDa) is indicated. Required parameters are missing or incorrect.

Incubation of HA with high concentrations of procyanidin B2-di-gallate (**8**) (1.13 mM) and EGCG (**6**) (2.18 mM) led to a time dependent slight reduction of the monomeric HA band and the appearance of HA aggregates being visible in Coomassie-stained gels as a broad 75 to>200 kDa “smear” (Figure 6A, C). After incubation of HA with EGCG (**6**) for 4 h to 24 h a faint band corresponding to HA dimers became visible (Figure 6A). Higher oligomers of HA could not be detected. As compared to mock treated HA, incubation with the galloylated oligomeric proanthocyanidins (**6**) and (**8**) only led to a moderate decrease in the intensity of the band corresponding to monomeric HA in Coomassie-stained gels (Figure 6A, C). Both compounds, however, reduced the strength of the HA monomer-specific signal in immunoblot (Figure 6B, D). The decrease in immunoreactivity of HA appeared to be more pronounced for (**6**).

Thus, (**6)** and (**8)** exhibit tannin-like astringent effects on HA when applied for prolonged times at high concentrations, i.e., at concentrations approx. 100 to 10,000-fold higher than the respective IC_50_ values in MTT_IAV_ assay and plaque reduction assay, respectively. The observed “smear” in SDS-PAGE and immunoblots may stem from HA literally coated with various amounts of (**6)** and (**8)**. This may also account for the reduced reactivity of the His-tag-specific monoclonal antibody used to detect recombinant soluble HA. Similar effects were observed with an HA-specific monoclonal antibody (data not shown). The effects of high concentrations of extract RA and its active compounds on HA are in good accordance with the model suggested by Haslam (1996) [45] by describing the aggregation of proteins by polyphenols, and confirms earlier findings in HSV-1 [21]. On the other hand, antiviral effects of (**6)** and (**8)** are detectable at much lower concentrations. Therefore, similar to what was observed for RA, tannin-like astringent effects are unlikely to mediate most of the antiviral activity of these compounds.

### Procyanidin B2-di-gallate (8) is predicted *in silico* to interact with the sialic acid binding site of viral hemagglutinin

**Figure 7:**
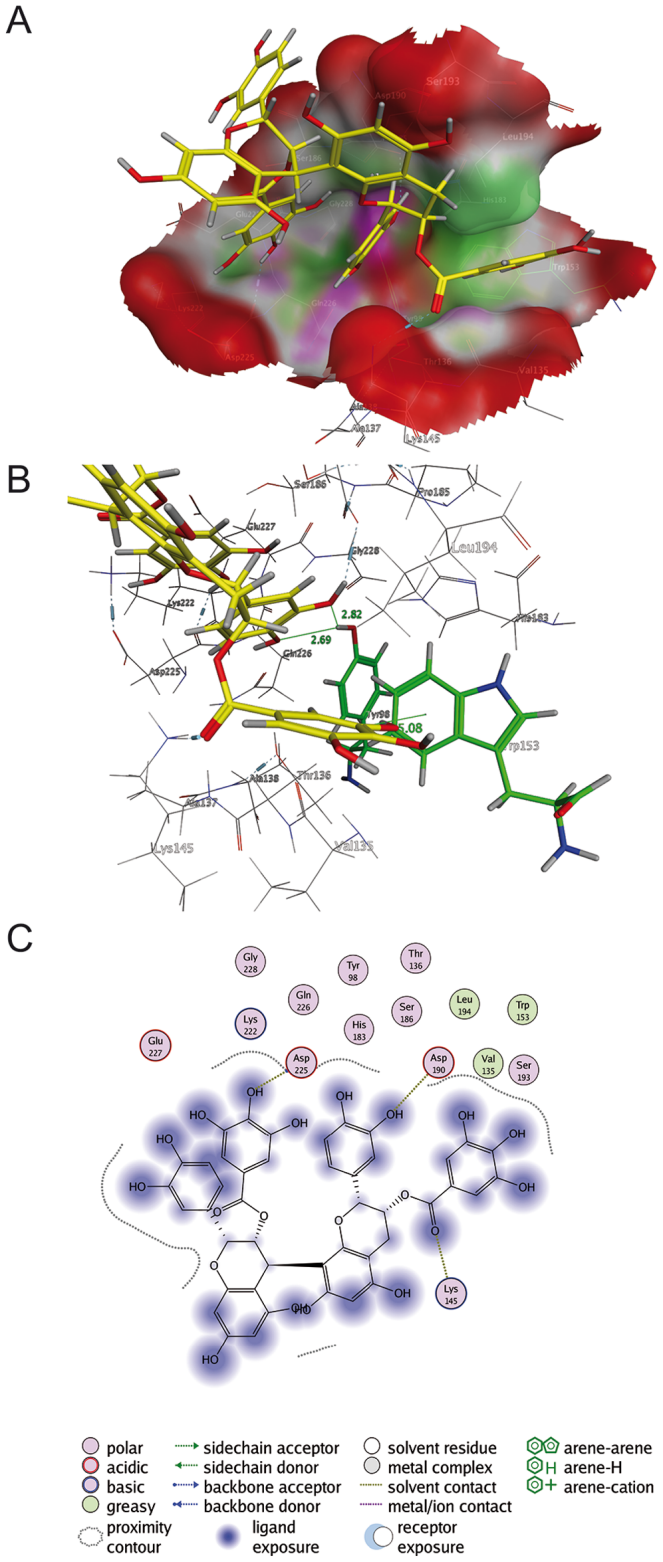
Protein-ligand docking of epicatechin-3-O-gallate-(4β→8)-epicatechin-3′-O-gallate (8) into the sialic acid binding cavity of hemagglutinin. (A) 3D model; protein: green: hydrophobic, purple: polar, red: exposed; ligand: yellow: carbon, light grey: hydrogen, red: oxygen, blue: nitrogen; (B) Interactions of Tyr98 and Trp153; (C) 2D.

To visualize the binding of components from RA to the viral surface proteins, four selected compounds were docked to HA of influenza virus A/California/04/2009 (H1N1) [30] *in silico* by means of the software package MOE. Exemplary for the docking results of all investigated cavities of HA, the score of the docking at the sialic acid binding site [46] was -6.29 for procyanidin B2-di-gallate (**8**), −5.55 for procyanidin B2 (**7**), −5.89 for EGCG (**6**), and −5.28 for epicatechin (**2**), with (**8**) showing the best score. The data demonstrated a better score of galloylated compounds in comparison to the respective ungalloylated molecules. Additionally, inspection of the best docking pose revealed the binding of (**8**) (Figure 7) with both galloyl moieties and the B-ring of the second epicatechin gallate unit deep inside the sialic acid binding pocket, suggesting a notably stronger anchorage of galloylated molecules in contrast to ungalloylated compounds and offering a straightforward explanation for the strong activity of this digalloylated dimer. Aside from this, the investigated dimers (**7**) and (**8**) yielded a better docking score than the monomeric (**6**) and (**2**). These results further corroborate the observation depicted in the functional bioassays: An increase in the degree of polymerization and galloylation enhances the binding of proanthocyanidins to HA. As discussed already above, these results are in contrast to a model favoring the unspecific “coating” of HA by polyphenols. The strong anchoring of the galloylated compounds (**6**) and (**8**) in the sialic acid binding pocket of HA disclosed by *in silico* visualization may block the receptor binding site of HA and consequently specifically inhibit the viral adsorption process.

EGCG (**6**) blocks binding of HIV gp120 to its cellular receptor CD4, and it has been suggested that there is an appropriate binding site of EGCG (**6**) in the region of CD4 interacting with gp120. The galloyl ring D of EGCG (**6**) appears to stack against aromatic and basic amino acid side chains within the gp120 binding site of CD4, e.g., Phe 43, Arg 59, Trp62 of CD4, thereby abrogating interaction of gp120 with CD4 [47]. Notably, crystal structure analyses revealed that a subgroup of neutralizing antibodies interferes with receptor binding of HA by targeting the highly conserved Tyr98 and Trp153 at the hydrophobic cavity base of the sialic acid binding site with an aromatic side chain [48], [49]. It is therefore worth mentioning that in our docking model, the galloyl moiety of the second epicatechin gallate unit of procyanidin B2-di-gallate (**8**) is close to the aromatic side chain of Trp153 in the sialic acid binding pocket of HA, where it might interact in terms of a T-shaped π-π interaction. Furthermore, the B-ring of the second subunit is in a position where its phenolic oxygens might form hydrogen bonds with the hydroxyl proton of Tyr98 (both distances O…H<3 Å; see Figure 7B).

### RA does not interfere with cellular responses to TNF-α and EGF

**Figure 8:**
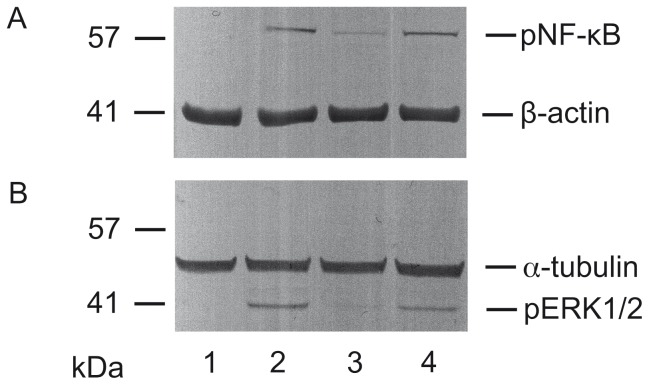
Influence of extract RA on TNF-α (A) and EGF (B) induced signal transduction in A549 cells. Lanes 1 and 2 represent cells preincubated for 1 h with medium, lanes 3 and 4 with RA (100 µg/mL). (**A**) Stimulation of cells with TNF-α (20 ng/mL, 30 min.) (lane 2, 4), and detection of phosphorylated NF-κB; loading control β-actin; (**B**) stimulation of cells with EGF (30 ng/mL, 10 min.) (lane 2, 4), and detection of phosphorylated ERK1/2; loading control α-tubulin.

While the extract RA showed little cytotoxic effect over a wide range of concentrations it might still elicit or interfere with intracellular responses in treated cells. Thus, the effect of the addition of high concentrations of RA (100 µg/mL) close to the calculated CC_50_ for 1 h at 37°C on TNF-α and EGF induced signal transduction was studied. As shown in Figure 8A stimulation of A549 cells by TNF-α led to similar increases in phosphorylated NF-κB (pNF-κB) in RA-treated or mock-treated cells, respectively. In the absence of TNF-α, neither RA nor mock-treatment led to a significant induction of pNF-κB. Potential effects of RA on Raf/MEK/ERK-signaling were investigated by stimulation of A549 cells by EGF (Figure 8B). While non-EGF-stimulated cells did not express pERK1/2, regardless if pretreated with RA or not (lanes 1 and 3), EGF treatment activated its expression (lane 2). Pretreatment of the cells with RA, followed by stimulation with EGF did not result in a significant decrease in pERK1/2 expression. It was thus concluded that even high concentrations of RA close to the CC_50_ are unlikely to significantly elicit or interfere with TNF-α and EGF-induced signal transduction. This is in accordance to recent results showing that cells are inert to LADANIA067, a polyphenol-rich extract of *Ribes nigrum folium* inhibiting entry of IAV [50].

## Conclusions

The proanthocyanidin-enriched extract RA and its main active constituent epicatechin-3-O-gallate-(4β→8)-epicatechin-3′-O-gallate (procyanidin B2-di-gallate) protect cells from IAV infection by blocking IAV adsorption and interfering with penetration at higher concentrations. Anti-IAV-activity is dependent on galloylation of the procyanidin backbone. At effective concentrations, cells are unaffected by RA and procyanidin B2-di-gallat. Regarding the need for new and abundantly available anti-influenza therapeutics, RA and procyanidin B2-di-gallate appear to be a promising expansion of the currently available anti-influenza agents.

## Supporting Information

Figure S1 Inhibitory effect of residual allantoic fluid on the antiviral activity of RA. To demonstrate that titres of viral stocks prepared from allantoic fluid of infected eggs have an impact on the outcome of the MTT_IAV_ assay, stocks of isolate I1 (H1N1)pdm09 were approx. 50-fold prediluted in allantoic fluid (from 3.2×10^8^ pfu/mL to 6.6×10^6^ pfu/mL). Subsequently, virus was diluted to 1×10^4^ pfu IAV/well in serum-free medium and the antiviral activity and cell vitality were determined by MTT_IAV_ assay and cytotoxicity assay, respectively (compare Figure 2). Values represent mean ±SD of ≥3 independent experiments, * p<0.05, ** p<0.01 (two-tailed, unpaired Student's t-test). Statistical significance of antiviral activity was calculated for nontoxic concentrations only (1 to 5 µg/mL).
(TIF)

## References

[1] Lambert LC, Fauci AS (2010) Influenza vaccines for the future. N Engl J Med 363: 2036–2044. 21083388 10.1056/NEJMra1002842

[2] Taubenberger JK, Morens DM (2006) 1918 Influenza: the mother of all pandemics. Emerg Infect Dis 12: 15–22. 16494711 10.3201/eid1201.050979 PMC3291398

[3] Kamali A, Holodniy M (2013) Influenza treatment and prophylaxis with neuraminidase inhibitors: a review. Infect Drug Resist 6: 187–198. 24277988 10.2147/IDR.S36601 PMC3838482

[4] Fiore AE, Fry A, Shay D, Gubareva L, Bresee JS, et al (2011) Antiviral agents for the treatment and chemoprophylaxis of influenza --- recommendations of the Advisory Committee on Immunization Practices (ACIP). MMWR Recomm Rep 60: 1–24. 21248682

[5] Thorlund K, Awad T, Boivin G, Thabane L (2011) Systematic review of influenza resistance to the neuraminidase inhibitors. BMC Infect Dis 11: 134. 21592407 10.1186/1471-2334-11-134 PMC3123567

[6] Dixit R, Khandaker G, Ilgoutz S, Rashid H, Booy R (2013) Emergence of oseltamivir resistance: control and management of influenza before, during and after the pandemic. Infect Disord Drug Targets 13: 34–45. 23675925 10.2174/18715265112129990006

[7] De Clercq E (2013) Antivirals: past, present and future. Biochem Pharmacol 85: 727–744. 23270991 10.1016/j.bcp.2012.12.011

[8] De Clercq E (2013) A Cutting-Edge View on the Current State of Antiviral Drug Development. Med Res Rev 33: 1249–1277. 23495004 10.1002/med.21281

[9] Laursen NS, Wilson IA (2013) Broadly neutralizing antibodies against influenza viruses. Antiviral Res 98: 476–483. 23583287 10.1016/j.antiviral.2013.03.021 PMC3987986

[10] Wang X, Jia W, Zhao A, Wang X (2006) Anti-influenza agents from plants and traditional Chinese medicine. Phytother Res 20: 335–341. 16619359 10.1002/ptr.1892

[11] Ge H, Wang YF, Xu J, Gu Q, Liu HB, et al (2010) Anti-influenza agents from Traditional Chinese Medicine. Nat Prod Rep 27: 1758–1780. 20941447 10.1039/c0np00005a

[12] Jassim SA, Naji MA (2003) Novel antiviral agents: a medicinal plant perspective. J Appl Microbiol 95: 412–427. 12911688 10.1046/j.1365-2672.2003.02026.x

[13] Daglia M (2012) Polyphenols as antimicrobial agents. Curr Opin Biotechnol 23: 174–181. 21925860 10.1016/j.copbio.2011.08.007

[14] Song JM, Seong BL (2007) Tea catechins as a potential alternative anti-infectious agent. Expert Rev Anti Infect Ther 5: 497–506. 17547513 10.1586/14787210.5.3.497

[15] Steinmann J, Buer J, Pietschmann T, Steinmann E (2013) Anti-infective properties of epigallocatechin-3-gallate (EGCG), a component of green tea. Br J Pharmacol 168: 1059–1073. 23072320 10.1111/bph.12009 PMC3594666

[16] Yang ZF, Bai LP, Huang WB, Li XZ, Zhao SS, et al (2014) Comparison of in vitro antiviral activity of tea polyphenols against influenza A and B viruses and structure-activity relationship analysis. Fitoterapia 93: 47–53. 24370660 10.1016/j.fitote.2013.12.011

[17] Nakayama M, Suzuki K, Toda M, Okubo S, Hara Y, et al (1993) Inhibition of the infectivity of influenza virus by tea polyphenols. Antiviral Res 21: 289–299. 8215301 10.1016/0166-3542(93)90008-7

[18] Imanishi N, Tuji Y, Katada Y, Maruhashi M, Konosu S, et al (2002) Additional inhibitory effect of tea extract on the growth of influenza A and B viruses in MDCK cells. Microbiol Immunol 46: 491–494. 12222936 10.1111/j.1348-0421.2002.tb02724.x

[19] Song JM, Lee KH, Seong BL (2005) Antiviral effect of catechins in green tea on influenza virus. Antiviral Res 68: 66–74. 16137775 10.1016/j.antiviral.2005.06.010

[20] Ubillas R, Jolad SD, Bruening RC, Kernan MR, King SR, et al (1994) SP-303, an antiviral oligomeric proanthocyanidin from the latex of Croton lechleri (Sangre de Drago). Phytomedicine 1: 77–106. 23195881 10.1016/S0944-7113(11)80026-7

[21] Gescher K, Hensel A, Hafezi W, Derksen A, Kuhn J (2011) Oligomeric proanthocyanidins from Rumex acetosa L. inhibit the attachment of herpes simplex virus type-1. Antiviral Res 89: 9–18. 21070811 10.1016/j.antiviral.2010.10.007

[22] Gescher K, Kuhn J, Lorentzen E, Hafezi W, Derksen A, et al (2011) Proanthocyanidin-enriched extract from Myrothamnus flabellifolia Welw. exerts antiviral activity against herpes simplex virus type 1 by inhibition of viral adsorption and penetration. J Ethnopharmacol 134: 468–474. 21211557 10.1016/j.jep.2010.12.038

[23] Glatthaar-Saalmuller B, Rauchhaus U, Rode S, Haunschild J, Saalmuller A (2011) Antiviral activity in vitro of two preparations of the herbal medicinal product Sinupret(R) against viruses causing respiratory infections. Phytomedicine 19: 1–7. 22112724 10.1016/j.phymed.2011.10.010 PMC7125718

[24] Bicker J, Petereit F, Hensel A (2009) Proanthocyanidins and a phloroglucinol derivative from Rumex acetosa L. Fitoterapia. 80: 483–495. 10.1016/j.fitote.2009.08.015 19695312

[25] Pabst D, Kuehn J, Schuler-Luettmann S, Wiebe K, Lebiedz P (2011) Acute Respiratory Distress Syndrome as a presenting manifestation in young patients infected with H1N1 influenza virus. Eur J Intern Med 22: e119–124. 22075296 10.1016/j.ejim.2011.08.014

[26] Hrincius ER, Wixler V, Wolff T, Wagner R, Ludwig S, et al (2010) CRK adaptor protein expression is required for efficient replication of avian influenza A viruses and controls JNK-mediated apoptotic responses. Cell Microbiol 12: 831–843. 20088952 10.1111/j.1462-5822.2010.01436.x

[27] Mosmann T (1983) Rapid colorimetric assay for cellular growth and survival: application to proliferation and cytotoxicity assays. J Immunol Methods 65: 55–63. 6606682 10.1016/0022-1759(83)90303-4

[28] Beyleveld G, White KM, Ayllon J, Shaw ML (2013) New-generation screening assays for the detection of anti-influenza compounds targeting viral and host functions. Antiviral Res 100: 120–132. 23933115 10.1016/j.antiviral.2013.07.018 PMC3840122

[29] Pauwels R, Balzarini J, Baba M, Snoeck R, Schols D, et al (1988) Rapid and automated tetrazolium-based colorimetric assay for the detection of anti-HIV compounds. J Virol Methods 20: 309–321. 2460479 10.1016/0166-0934(88)90134-6

[30] Xu R, Ekiert DC, Krause JC, Hai R, Crowe JE Jr, et al (2010) Structural basis of preexisting immunity to the 2009 H1N1 pandemic influenza virus. Science 328: 357–360. 20339031 10.1126/science.1186430 PMC2897825

[31] Pariani E, Amendola A, Ranghiero A, Anselmi G, Zanetti A (2013) Surveillance of influenza viruses in the post-pandemic era (2010-2012) in Northern Italy. Hum Vaccin Immunother 9. 10.4161/hv.23262 PMC3891725 23302775

[32] Khandaker I, Suzuki A, Kamigaki T, Tohma K, Odagiri T, et al.. (2013) Molecular evolution of the hemagglutinin and neuraminidase genes of pandemic (H1N1) 2009 influenza viruses in Sendai, Japan, during 2009–2011. Virus Genes. 10.1007/s11262-013-0980-5 PMC3834170 24078044

[33] Zhang JH, Chung TD, Oldenburg KR (1999) A Simple Statistical Parameter for Use in Evaluation and Validation of High Throughput Screening Assays. J Biomol Screen 4: 67–73. 10838414 10.1177/108705719900400206

[34] Theisen LL, Muller CP (2012) EPs(R) 7630 (Umckaloabo(R)), an extract from Pelargonium sidoides roots, exerts anti-influenza virus activity in vitro and in vivo. Antiviral Res 94: 147–156. 22475498 10.1016/j.antiviral.2012.03.006

[35] De Bruyne T, Pieters L, Witvrouw M, De Clercq E, Vanden Berghe D, et al (1999) Biological evaluation of proanthocyanidin dimers and related polyphenols. J Nat Prod 62: 954–958. 10425115 10.1021/np980481o

[36] Zumdick S, Deters A, Hensel A (2012) In vitro intestinal transport of oligomeric procyanidins (DP 2 to 4) across monolayers of Caco-2 cells. Fitoterapia 83: 1210–1217. 22776719 10.1016/j.fitote.2012.06.013

[37] Wang R, Lechtenberg M, Sendker J, Petereit F, Deters A, et al (2013) Wound-healing plants from TCM: in vitro investigations on selected TCM plants and their influence on human dermal fibroblasts and keratinocytes. Fitoterapia 84: 308–317. 23266731 10.1016/j.fitote.2012.12.020

[38] Agyare C, Lechtenberg M, Deters A, Petereit F, Hensel A (2011) Ellagitannins from Phyllanthus muellerianus (Kuntze) Exell.: Geraniin and furosin stimulate cellular activity, differentiation and collagen synthesis of human skin keratinocytes and dermal fibroblasts. Phytomedicine 18: 617–624. 21036574 10.1016/j.phymed.2010.08.020

[39] Park S, Kim JI, Lee I, Lee S, Hwang MW, et al (2013) Aronia melanocarpa and its components demonstrate antiviral activity against influenza viruses. Biochem Biophys Res Commun 440: 14–19. 24012672 10.1016/j.bbrc.2013.08.090

[40] Liu G, Xiong S, Xiang YF, Guo CW, Ge F, et al (2011) Antiviral activity and possible mechanisms of action of pentagalloylglucose (PGG) against influenza A virus. Arch Virol 156: 1359–1369. 21479599 10.1007/s00705-011-0989-9

[41] Theisen LL, Erdelmeier CA, Spoden GA, Boukhallouk F, Sausy A, et al (2014) Tannins from Hamamelis virginiana Bark Extract: Characterization and Improvement of the Antiviral Efficacy against Influenza A Virus and Human Papillomavirus. PLoS One 9: e88062. 24498245 10.1371/journal.pone.0088062 PMC3909258

[42] Ehrhardt C, Hrincius ER, Korte V, Mazur I, Droebner K, et al (2007) A polyphenol rich plant extract, CYSTUS052, exerts anti influenza virus activity in cell culture without toxic side effects or the tendency to induce viral resistance. Antiviral Res 76: 38–47. 17572513 10.1016/j.antiviral.2007.05.002

[43] Droebner K, Ehrhardt C, Poetter A, Ludwig S, Planz O (2007) CYSTUS052, a polyphenol-rich plant extract, exerts anti-influenza virus activity in mice. Antiviral Res 76: 1–10. 17573133 10.1016/j.antiviral.2007.04.001

[44] Haidari M, Ali M, Ward Casscells S 3rd, Madjid M (2009) Pomegranate (Punica granatum) purified polyphenol extract inhibits influenza virus and has a synergistic effect with oseltamivir. Phytomedicine 16: 1127–1136. 19586764 10.1016/j.phymed.2009.06.002

[45] Haslam E (1996) Natural polyphenols (vegetable tannins) as drugs: possible modes of action. J Nat Prod 59: 205–215. 8991956 10.1021/np960040+

[46] Sauter NK, Hanson JE, Glick GD, Brown JH, Crowther RL, et al (1992) Binding of influenza virus hemagglutinin to analogs of its cell-surface receptor, sialic acid: analysis by proton nuclear magnetic resonance spectroscopy and X-ray crystallography. Biochemistry 31: 9609–9621. 1327122 10.1021/bi00155a013

[47] Williamson MP, McCormick TG, Nance CL, Shearer WT (2006) Epigallocatechin gallate, the main polyphenol in green tea, binds to the T-cell receptor, CD4: Potential for HIV-1 therapy. J Allergy Clin Immunol 118: 1369–1374. 17157668 10.1016/j.jaci.2006.08.016

[48] Xu R, Krause JC, McBride R, Paulson JC, Crowe JE Jr, et al (2013) A recurring motif for antibody recognition of the receptor-binding site of influenza hemagglutinin. Nat Struct Mol Biol 20: 363–370. 23396351 10.1038/nsmb.2500 PMC3594569

[49] Ekiert DC, Kashyap AK, Steel J, Rubrum A, Bhabha G, et al (2012) Cross-neutralization of influenza A viruses mediated by a single antibody loop. Nature 489: 526–532. 22982990 10.1038/nature11414 PMC3538848

[50] Ehrhardt C, Dudek SE, Holzberg M, Urban S, Hrincius ER, et al (2013) A plant extract of Ribes nigrum folium possesses anti-influenza virus activity in vitro and in vivo by preventing virus entry to host cells. PLoS One 8: e63657. 23717460 10.1371/journal.pone.0063657 PMC3662772

[51] Danne A, Petereit F, Nahrstedt A (1994) Flavan-3-ols, prodelphinidins and further polyphenols from Cistus salvifolius. Phytochemistry 37: 533–538. 7765630 10.1016/0031-9422(94)85094-1

