# Supplementary figures and images for: 3-O-Galloylated Procyanidins from Rumex acetosa L. Inhibit the Attachment of Influenza A Virus
Source: PLoS One. 2014 Oct 10;9(10):e110089. doi: 10.1371/journal.pone.0110089 (PMC4193892; doi:10.1371/journal.pone.0110089)

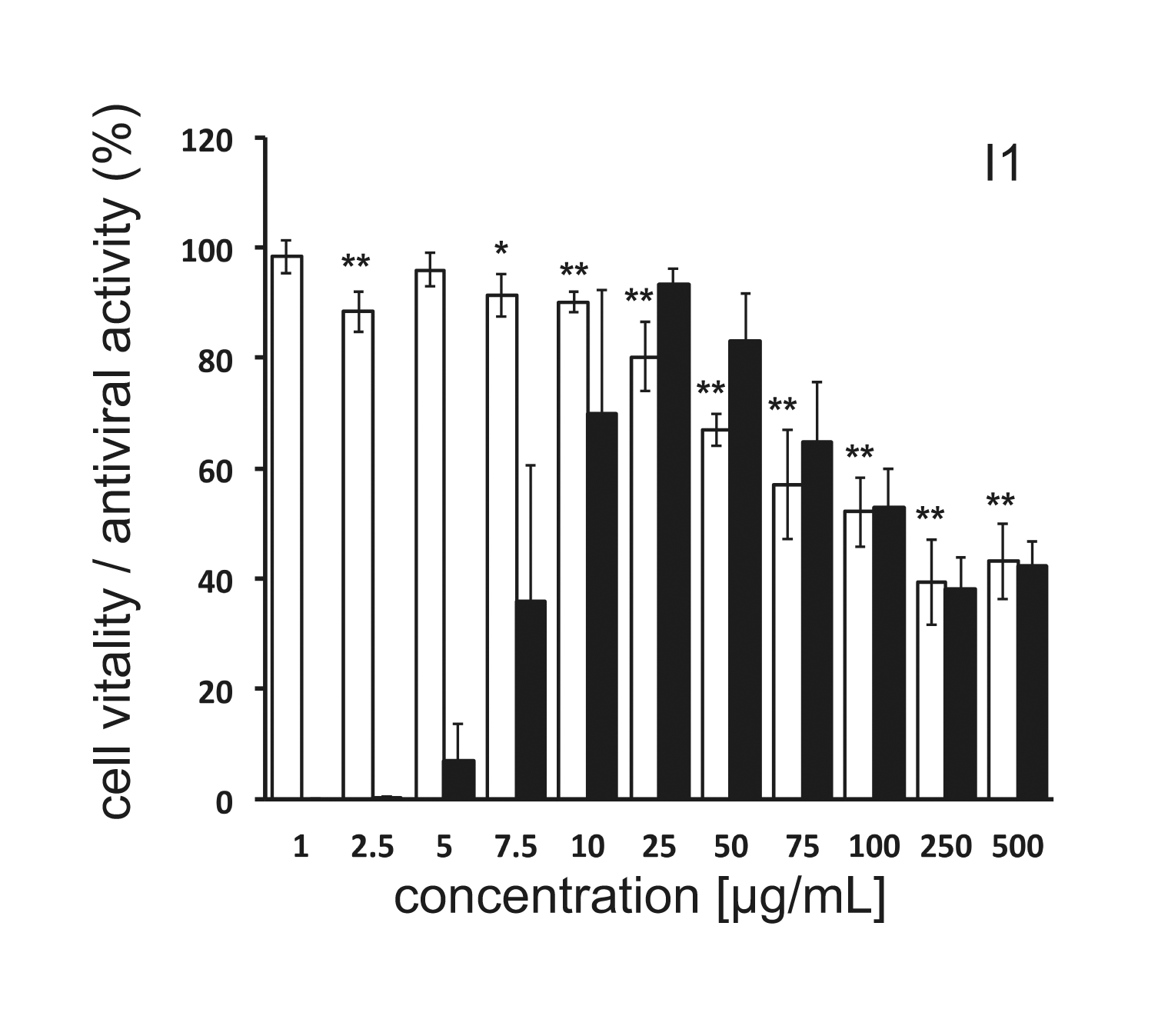

Supplement: Figure S1 — Inhibitory effect of residual allantoic fluid on the antiviral activity of RA. To demonstrate that titres of viral stocks prepared from allantoic fluid of infected eggs have an impact on the outcome of the MTTIAV assay, stocks of isolate I1 (H1N1)pdm09 were approx. 50-fold prediluted in allantoic fluid (from 3.2×10^8^ pfu/mL to 6.6×10^6^ pfu/mL). Subsequently, virus was diluted to 1×10^4^ pfu IAV/well in serum-free medium and the antiviral activity and cell vitality were determined by MTTIAV assay and cytotoxicity assay, respectively (compare Figure 2). Values represent mean ±SD of ≥3 independent experiments, * p<0.05, ** p<0.01 (two-tailed, unpaired Student's t-test). Statistical significance of antiviral activity was calculated for nontoxic concentrations only (1 to 5 µg/mL). (TIF) [file pone.0110089.s001.tif]
